# Racial Disparities in Donor Human Milk Feedings: A Study Using Electronic Medical Records

**DOI:** 10.1089/heq.2022.0085

**Published:** 2022-10-19

**Authors:** Aunchalee E.L. Palmquist, Ifeyinwa V. Asiodu, Christine Tucker, Kristin P. Tully, Diane T. Asbill, Angela Malloy, Alison M. Stuebe

**Affiliations:** ^1^ Department of Maternal and Child Health, Gillings School of Global Public Health, University of North Carolina at Chapel Hill, Chapel Hill, North Carolina, USA.; ^2^ Carolina Population Center, University of North Carolina at Chapel Hill, Chapel Hill, North Carolina, USA.; ^3^ Department of Family Health Care Nursing, University of California, San Francisco, California, USA.; ^4^ Department of Obstetrics and Gynecology, School of Medicine, University of North Carolina at Chapel Hill, Chapel Hill, North Carolina, USA.; ^5^ N.C. Women's Hospital, UNC Chapel Hill, North Carolina, USA.; ^6^ Momma's Village of Fayetteville, Fayetteville, North Carolina, USA.

**Keywords:** breastfeeding, donor human milk, electronic medical records, neonatal intensive care unit, racism

## Abstract

**Introduction:**

The aim of this study was to evaluate differences in the use of pasteurized donor human milk (PDHM) by maternal race–ethnicity during postpartum hospitalization using electronic medical records (EMRs).

**Materials and Methods:**

A retrospective cohort study of all live-born infants at our academic research institution from July 1, 2014, to June 30, 2016, was conducted. EMR data were used to determine whether each infant received mother's own milk (MOM), PDHM, or formula. These data were stratified based on whether the infant received treatment in the Neonatal Critical Care Center. Generalized estimating equation models were used to calculate the odds of receiving PDHM by maternal race–ethnicity, adjusting for gestational age, birth weight, insurance, preferred language, nulliparity, and mode of delivery.

**Results:**

Infant feeding data were available for 7097 infants, of whom 49% were fed only MOM during their postpartum hospitalization. Among the 15.9% of infants admitted to neonatal critical care, infants of non-Hispanic Black (odds ratio [OR] 0.47, 95% confidence interval [CI] 0.31–0.72), Hispanic (OR 0.65, 95% CI 0.36–1019), and Other (OR 0.63, 95% CI 0.32–1.26) mothers had lower rates of PDHM feedings than infants of non-Hispanic White mothers in the adjusted models. Among well infants, the use of PDHM was lower among non-Hispanic Black and Hispanic mothers (OR 0.25, 95% CI 0.18–0.36, and OR 0.38, 95% CI 0.26–0.56) compared with non-Hispanic White mothers.

**Conclusions:**

Inequities in exclusive human milk feeding and use of PDHM by maternal race–ethnicity were identified. Antiracist interventions are needed to promote equitable access to skilled lactation support and counseling for PDHM use.

## Introduction

Breastfeeding is vital to maternal and infant health.^1^ The American Academy of Pediatrics (AAP),^2,3^ Academy of Breastfeeding Medicine (ABM),^4^ and Association of Women's Health, Obstetric, and Neonatal Nurses (AWHONN)^5^ recommend exclusive breastfeeding during the first 6 months of life. While most women in the United States initiate breastfeeding immediately postpartum, there are significant racial disparities in both breastfeeding initiation and other longer-term breastfeeding outcomes.^6,7^

A critical understanding of structural racism and its effects on perinatal health is essential to making sense of racial disparities in infant feeding practices.^8–11^ While intersecting social determinants of health that may circumscribe population-based group differences in infant feeding practices,^12^ racialized breastfeeding disparities in the United States are fundamentally tied to structural racism.^6,13,14^

Racism is experienced during postpartum hospitalization as discrimination, stigmatization, being stereotyped, criminalization, and violence,^15–23^ along with exposure to clinical practices and interactions that violate patient safety, autonomy, and dignity.^10^ Racism is often at the root of negative attitudes about breastfeeding or a preference for formula feeding in communities of color.^24^

For example, the historical context of chattel slavery in the United States is tied up with negative associations of breastfeeding with enslavement, coerced wet-nursing, and obstetric violence in some Black communities.^25,26^ Black feminist scholars, in particular, have described in their research the multitude of ways that historical, structural, institutional, and internalized racism is related to racialized breastfeeding disparities; they also lift up examples of Black women, their families, and communities overcoming racism and related barriers to breastfeeding.^6,14,25,27–30^

Racial disparities in lactation care and human milk feeding practices are especially pronounced in neonatal intensive care units (NICUs).^31–36^ For example, Black infants are twice as likely to be born prematurely (<37 weeks' gestation) and to require neonatal intensive care than non-Hispanic White infants.^37^ Exclusive human milk feedings for medically fragile preterm infants are critical for preventing necrotizing enterocolitis and related mortality, which is exacerbated by preterm formula feeding.^38^

However, premature birth is often associated with medical interventions that disrupt lactation and lead to a cascade of complex feeding challenges.^39–41^ When maternal milk is not available or if supplementation is required, pasteurized donor human milk (PDHM) is widely considered a standard of care.^2,3,38,42^ Use of PDHM as part of an integrated approach to lactation care helps to maintain exclusive human milk feeding in the immediate postnatal period and supports continued breastfeeding once supplementation is no longer needed.^43–45^

Optimal lactation outcomes in the NICU are best supported when postpartum patients receive timely skilled lactation support, mental health care, and psychosocial support, which facilitate provisioning of expressed milk and breastfeeding.^46,47^ Yet, numerous studies have demonstrated that Black mothers receive poorer quality lactation support during postpartum hospitalization and that Black infants in the NICU are less likely to be breastfed or receive PDHM as recommended.^35,48–52^

Increasingly, PDHM is offered as a supplement to term infants, often as a strategy to protect and promote exclusive human milk feeding during postpartum hospitalization.^53–56^ PDHM is compositionally more similar to raw maternal milk than formula and provides infants with essential human milk bioactives that support immune function, which are not available in any commercially available infant formula.^57^

Although there is limited evidence for feeding term infants with PDHM, use of PDHM for term infants is aligned with the recommendations of the World Health Organization (WHO),^58^ ABM,^59^ AWHONN,^5^ and AAP.^60^ Nonetheless, even in hospitals with policies that make it possible for well newborns to receive PDHM, there have been reports of racial and ethnic disparities in its use.^61,62^

Addressing racialized disparities in lactation and infant feeding support is critically important to addressing the Black maternal and infant health crisis in the United States.^6^ Previous research at our institution revealed racial and ethnic inequities in postpartum pain evaluation and management,^18^ which are consistent with broader trends in health care providers' neglect of Black maternal and infant pain and suffering.^32,63–65^

In light of the significance of lactation on maternal health outcomes,^66^ the critical importance of human milk in preventing adverse newborn outcomes in the NICU,^66,67^ and the importance of exclusive human milk feeding in the first 6 months of life, the objective of our study was to evaluate the use of PDHM at our institution. Our study examines whether differences in use of PDHM for infant feeding are stratified by race and ethnicity through an analysis of hospital electronic medical records (EMRs).

## Patients and Methods

The Biomedical Institutional Review Board of the University of North Carolina at Chapel Hill granted ethics approval for the study (no. 16-0980). A retrospective cohort study of all live-born infants at North Carolina Women's Hospital (NCWH) from July 1, 2014, through June 30, 2016, was conducted by examining patient data within the EMRs. NCWH is a large, Level IV, regional referral university-affiliated hospital that provides specialty prenatal, postpartum, and neonatal care, with 14 maternal–fetal medicine specialists and 11 neonatologists.

The Neonatal Critical Care Center (NCCC) admits more than 800 infants a year from 50 counties across the state. NCWH is a Baby-Friendly designated hospital. This analysis was part of a larger mixed methods study to explore the health care needs and experiences of postpartum patients with infants in the NCCC.^40,41^

Postpartum patients and their infants were identified through the hospital's perinatal database, which is maintained by trained nurse abstractors who review medical records for all births at the facility. Clinical information for postpartum patients was obtained from the perinatal database. The Carolina Data Warehouse for Health was used to access discrete data from the Epic EMR, including infant admission to intensive care, maternal race and ethnicity, primary language, and payer status.

Race and ethnicity as recorded in the EMR during patient registration were combined into a race–ethnicity variable. Racial categories included American Indian, Asian/Pacific Islander, Black, White, and Other. Ethnicity categories were Hispanic and non-Hispanic.

The category “Other” initially included records of patients whose race or ethnicity was reported as other, refused, or unknown. For the analysis, when a patient's race was reported as American Indian, they were grouped with Other due to stipulations by data warehouse administrators that reporting cell sizes of <10 were not permitted. The non-Hispanic White race–ethnicity category was selected as the referent during analyses due to the larger sample size that permitted more power to detect differences in use of PDHM by race–ethnicity.

All live-born infants who initiated feeding (*n*=7097) were included. Flowsheets from the EMR were used to create infant feeding in the hospital category variables (Table 1). Infants were assigned to the exclusive mother's own milk group (“Exclusive MOM”) if the EMR indicated that the infant was exclusively breastfed, fed exclusively with expressed MOM, or some combination. Infants who were breastfed and received any PDHM supplementation were assigned to the “Any PDHM and no formula” group.

**Table 1.** Sociodemographic Characteristics, Infant Feeding, and Perinatal Outcomes of Mothers of Live-Born Infants at NCWH Hospital, by Race and Ethnicity (*n*=7097)

	Total		Non-Hispanic White		Non-Hispanic Black		Non-Hispanic Asian/Pacific Islander		Hispanic		Other		Chi-square
	*n*	%	*n*	%	*n*	%	*n*	%	*n*	%	*n*	%	*p* ^*^
Characteristics													
Infant feeding in the hospital													<0.0001
Exclusive mother's own milk	3475	49.0	1920	60.5	424	34.1	168	53.2	679	37.9	284	49.9	
Any donor human milk/no formula	665	9.4	399	12.6	91	7.3	33	10.4	82	4.6	60	10.5	
Any donor human milk and any formula	384	5.4	178	5.6	103	8.3	11	3.5	68	3.8	24	4.2	
Mother's own milk and formula	1994	28.1	452	14.2	400	32.2	96	30.4	882	49.2	164	28.8	
Exclusive formula	579	8.2	226	7.1	226	18.2	8	2.5	82	4.6	37	6.5	
Admitted to the NCCC	1127	15.9	531	16.7	282	22.7	18	5.7	192	10.7	104	18.3	<0.0001
Maternal age (years)													<0.0001
<20	316	4.5	84	2.6	73	5.9	<10	1.3	131	7.3	24	4.2	
20–34	5140	72.4	2263	71.3	967	77.7	213	67.4	1263	70.4	434	76.3	
≥35	1641	23.1	828	26.1	204	16.4	99	31.3	399	22.3	111	19.5	
Gestational age (weeks)													<0.0001
<34	374	5.3	171	5.4	122	9.8	<10	0.9	43	2.4	35	6.2	
34–<37	581	8.2	284	8.9	110	8.8	12	3.8	133	7.4	42	7.4	
37–<39	1764	24.9	714	22.5	360	28.9	85	26.9	488	27.2	117	20.6	
39–<41	3584	50.5	1593	50.2	559	44.9	177	56.0	944	52.6	311	54.7	
≥41	794	11.2	413	13.0	93	7.5	39	12.3	185	10.3	64	11.2	
Mode of delivery													<0.0001
Vaginal delivery only	4764	67.1	2145	67.6	703	56.5	204	64.6	1335	74.5	377	66.3	
Any operative vaginal delivery	259	3.6	136	4.3	29	2.3	21	6.6	39	2.2	34	6.0	
Any cesarean delivery	2074	29.2	894	28.2	512	41.2	91	28.8	419	23.4	158	27.8	
Insurance at delivery													<0.0001
Private	3258	45.9	2099	66.1	414	33.3	246	77.8	235	13.1	264	46.4	
Public	3479	49.0	898	28.3	783	62.9	61	19.3	1469	81.9	268	47.1	
Military	222	3.1	152	4.8	35	2.8	<10	0.9	13	0.7	19	3.3	
Self or unknown	138	1.9	26	0.8	12	1.0	<10	1.9	76	4.2	18	3.2	
Primary language													<0.0001
English	5624	79.2	3150	99.2	1224	98.4	228	72.2	579	32.3	443	77.9	
Spanish	1220	17.2	<10	0.1	<10	0.1	0	0.0	1167	65.1	49	8.6	
Other/unknown/missing	253	3.6	22	0.7	19	1.5	88	27.8	47	2.6	77	13.5	
Parity													<0.0001
1	2677	37.7	1375	43.3	464	37.3	162	51.3	423	23.6	253	44.5	
2	2241	31.6	1076	33.9	403	32.4	107	33.9	476	26.5	179	31.5	
3	1234	17.4	464	14.6	217	17.4	37	11.7	435	24.3	81	14.2	
≥4	945	13.3	260	8.2	160	12.9	10	3.2	459	25.6	56	9.8	
Total	7097	100.0	3175	44.7	1244	17.5	316	4.5	1793	25.3	569	8.0	

Source: the UNC Perinatal Database and the Carolina Data Warehouse for Health from July 1, 2014, to June 30, 2016.

^*^ *p*-Value from the Pearson chi-square test.

*n*, frequency; NCCC, Neonatal Critical Care Center.

Infants who were not only breastfed but also received supplementation with a combination of PDHM and formula were assigned to the “Any formula and any PDHM” group. Infants who were fed a combination of MOM and formula, but did not receive PDHM, were assigned to the “MOM and formula” group. Finally, infants who were only fed with formula were assigned to the “Exclusive formula” group.

For the analyses, all models were stratified by whether the infant was admitted to the NCCC (yes or no). Stratifying facilitated analysis of the relationship between race–ethnicity and PDHM feedings in different postnatal care settings, which utilize different protocols, priorities, and practices regarding PDHM access and use (S. Meier, personal communication). Postpartum patients whose infants were admitted to the postpartum floor for palliative care and those whose infants died before discharge were excluded from analyses.

Pearson chi-square tests were run to assess differences in sociodemographic characteristics, perinatal outcomes, and infant feeding practices by maternal race–ethnicity. The analysis was stratified by whether the infant was admitted to the NCCC, as defined above. Among infants who were not exclusively fed MOM, a logistic regression was used to estimate the relationship between maternal race–ethnicity and PDHM use during the postpartum stay, adjusting for gestational age at delivery, birth weight, insurance, preferred language, nulliparity, and mode of delivery.

For these analyses, generalized estimating equations (GEEs) were used to account for correlated observations among siblings born during the study period.

## Results

During the study period, 6765 patients birthed a total of 7097 infants. There were 6458 patients who had one pregnancy with a singleton birth, 115 who had two pregnancies with singleton births, 178 who had one pregnancy resulting in twins, 11 who had a single pregnancy resulting in triplets, and 3 who had two pregnancies—one resulting in a singleton and one in a set of twins.

Most births were vaginal deliveries (70.7%). Cesarean deliveries were highest among non-Hispanic Black patients and lowest among Hispanic patients (Table 1). Nearly 16% of infants had an NCCC stay, and the percent differed significantly by race–ethnicity, with the highest percent (23%) among infants of non-Hispanic Black mothers.

Nearly 92% of infants received any human milk and 49% received MOM exclusively. The percentage of infants fed with MOM varied widely by race–ethnicity from 34% to 61% (Table 1). Infants whose maternal race–ethnicity was non-Hispanic Black, Asian/Pacific Islander, Hispanic, or Other had lower rates of exclusive MOM feeding, lower PDHM supplementation, and higher formula feeding than infants whose maternal race–ethnicity was non-Hispanic White.

Exclusive formula feeding rates during postpartum hospitalization were significantly lower than all other feeding modes. However, infants whose maternal race–ethnicity was non-Hispanic Black were more likely to be given only formula (18.2%) than infants whose maternal race was reported as any other category.

Table 2 shows the distribution of all covariates by infant feeding category. There were statistically significant differences for all variables by feeding category, including NCCC admissions. NCCC stay was less prevalent among infants fed exclusively with MOM (3%) compared with infants in other feeding categories (Table 2). Infants fed MOM were more likely to have been born vaginally to mothers with private insurance, who were married or partnered, and who reported English as their preferred language.

**Table 2.** Sociodemographic Characteristics and Perinatal Outcomes of Mothers of Live-Born Infants at NCWH Hospital, by Infant Feeding Status in the Hospital (*n*=7097)

Characteristics	Exclusive MOM		Any PDHM and no formula		MOM and formula		Any formula and any PDHM		Exclusive formula		*p* ^*^
	*n*	%	*n*	%	*n*	%	*n*	%	*n*	%	
Admitted to the NCCC	115	3.3	268	40.3	297	77.3	326	16.3	121	20.9	<0.0001
Maternal race/ethnicity											<0.0001
Non-Hispanic White	1920	55.3	399	60.0	178	46.4	452	22.7	226	39.0	
Non-Hispanic Black	424	12.2	91	13.7	103	26.8	400	20.1	226	39.0	
Non-Hispanic Asian/Pacific Islander	168	4.8	33	5.0	11	2.9	96	4.8	<10	1.4	
Hispanic	679	19.5	82	12.3	68	17.7	882	44.2	82	14.2	
Other	284	8.2	60	9.0	24	6.3	164	8.2	37	6.4	
Maternal age (years)											<0.0001
<20	125	3.6	20	3.0	24	6.3	121	6.1	26	4.5	
20–34	2567	73.9	463	69.6	251	65.4	1418	71.1	441	76.2	
≥35	783	22.5	182	27.4	109	28.4	455	22.8	112	19.3	
Gestational age (weeks)											<0.0001
<34	17	0.5	132	19.8	172	44.8	38	1.9	15	2.6	
34–<37	74	2.1	130	19.5	89	23.2	218	10.9	70	12.1	
37–<39	811	23.3	155	23.3	57	14.8	554	27.8	187	32.3	
39–<41	2092	60.2	191	28.7	46	12.0	991	49.7	264	45.6	
≥41	481	13.8	57	8.6	20	5.2	193	9.7	43	7.4	
Mode of delivery											
Vaginal delivery only	2762	79.5	276	41.5	148	38.5	1219	61.1	359	62.0	
Any operative vaginal delivery	131	3.8	37	5.6	10	2.6	71	3.6	10	1.7	
Any cesarean section	582	16.7	352	52.9	226	58.9	704	35.3	210	36.3	
Insurance at delivery											<0.0001
Private	2036	58.6	418	62.9	174	45.3	510	25.6	120	20.7	
Public	1244	35.8	208	31.3	199	51.8	1390	69.7	438	75.6	
Military	128	3.7	31	4.7	9	2.3	43	2.2	11	1.9	
Self or unknown	67	1.9	<10	1.2	<10	0.5	51	2.6	10	1.7	
Primary language											<0.0001
English	2925	84.2	600	90.2	331	86.2	1252	62.8	516	89.1	
Spanish	440	12.7	41	6.2	45	11.7	642	32.2	52	9.0	
Other/unknown/missing	110	3.2	24	3.6	<10	2.1	100	5.0	11	1.9	
Parity											<0.0001
1	1416	40.7	379	57.0	179	46.6	572	28.7	131	22.6	
2	1200	34.5	164	24.7	107	27.9	584	29.3	186	32.1	
3	554	15.9	86	12.9	53	13.8	417	20.9	124	21.4	
≥4	305	8.8	36	5.4	45	11.7	421	21.1	138	23.8	
Total	3475	49.0	665	9.4	384	5.4	1994	28.1	579	8.2	

Source: the UNC Perinatal Database and the Carolina Data Warehouse for Health from July 1, 2014, to June 30, 2016.

^*^ *p*-Value from the Pearson chi-square test.

MOM, mother's own milk; PDHM, pasteurized donor human milk.

When stratified by NCCC admission, as expected, well infants who received standard care were more commonly fed exclusively with MOM, and among infants in the NCCC, feeding with any PDHM was more common (Table 3). The distribution of any PDHM feeding also varied by the length of NCCC stay. Among infants not admitted to the NCCC, use of any PDHM was highest among infants whose maternal race–ethnicity was non-Hispanic White or Asian/Pacific Islander and lowest among infants whose maternal race–ethnicity was non-Hispanic Black or Hispanic.

**Table 3.** Odds Ratios and 95% Confidence Intervals for Any Donor Human Milk Among Infants not Exclusively Breastfed While at NCWH Hospital, Stratified by Admission to Neonatal Critical Care (*n*=7097)

	*n*	Exclusive MOM	Any PDHM and no formula	Any formula and any PDHM	MOM and formula	Exclusive formula	Among not EBF, any PDHM	Any PDHM	Any PDHM
		%	%	%	%	%	%	Unadj OR (95% CI)	Adj^a^ OR (95% CI)
Not admitted to the NCCC									
Non-Hispanic White	2644	70.0	9.2	1.3	13.1	6.4	35.1	1.0 (ref)	1.0 (ref)
Non-Hispanic Black	962	43.1	3.6	1.9	32.3	19.0	9.7	0.20 (0.14–0.27)	0.25 (0.18–0.36)
Non-Hispanic Asian/Pacific Islander	298	55.0	10.1	1.7	30.5	2.7	26.1	0.65 (0.43–0.99)	0.53 (0.31–0.91)
Hispanic	1601	41.8	3.3	1.6	49.2	4.2	8.3	0.17 (0.13–0.22)	0.38 (0.26–0.56)
Other	465	56.3	7.7	0.9	28.6	6.5	19.7	0.45 (0.31–0.67)	0.60 (0.39–0.91)
Total	5970	3360	397	87	1668	458	484		
Admitted to the NCCC									
Non-Hispanic White	531	13.2	29.2	26.9	20.2	10.6	64.6	1.0 (ref)	1.0 (ref)
Non-Hispanic Black	282	3.2	19.9	30.1	31.6	15.3	51.7	0.58 (0.42–0.81)	0.47 (0.31–0.72)
Non-Hispanic Asian/Pacific Islander	18	22.2	16.7	33.3	27.8	0.0	64.3	0.98 (0.31–3.12)	1.04 (0.24–4.62)
Hispanic	192	5.2	15.6	22.4	49.0	7.8	40.1	0.37 (0.25–0.54)	0.65 (0.36–1.19)
Other	104	21.2	23.1	19.2	29.8	6.7	53.7	0.63 (0.38–1.07)	0.63 (0.32–1.26)
Total	1127	115	268	297	326	121	565		

Source: the UNC Perinatal Database and the Carolina Data Warehouse for Health from July 1, 2014, to June 30, 2016.

^a^ Adjusted for gestational age, primary language, insurance at delivery, mode of delivery, nulliparity, and birth weight.

CI, confidence interval; EBF, exclusively breastfed; OR, odds ratio.

There was less variation in PDHM use by maternal race–ethnicity among infants admitted to the NCCC. Infants of non-Hispanic White and Asian/Pacific Islander patients had the highest prevalence of any PDHM feedings (65% and 64%, respectively) compared with 52% and 40%, respectively, among infants of non-Hispanic Black and Hispanic patients.

In multivariable GEE models, these associations persisted. After adjusting for multiple variables, lower rates of any DHM feeding were found among infants in the NCCC whose maternal race–ethnicity was non-Hispanic Black, Hispanic, or Other; however, these estimates were only statistically significant for infants of non-Hispanic Black mothers (Table 3). These associations were unchanged when patients with a documented personal preference to combine breastfeeding and formula feeding were excluded from our analysis.

Among infants who were admitted to the NCCC and not fed exclusively with MOM, the use of PDHM varied by race–ethnicity. Lower rates of multivariable-adjusted PDHM use were found among infants whose maternal race–ethnicity was non-Hispanic Black, Hispanic and Asian/Pacific Islander compared with infants whose maternal race–ethnicity was non-Hispanic White, and all these estimates were statistically significant.

Among infants admitted to the NCCC, those covered by public insurance were less likely to receive any PDHM (odds ratio [OR] 0.37, 95% confidence interval [CI] 0.26–0.53) compared with those with private insurance. Similarly, among well infants, those covered by public insurance were less likely to receive any PDHM (OR 0.32, 95% CI 0.25–0.41) compared with those privately insured.

## Discussion

This retrospective analysis of EMR data revealed disparities in use of PDHM by race–ethnicity among well infants in the postnatal unit and among infants admitted to the NCCC. In addition, having public insurance was associated with reduced use of PDHM for supplemental feedings. These differences by race–ethnicity were not fully explained by clinical factors, language preference, or insurance type and persisted when the sample was restricted to postpartum patients without a documented preference of combining breastfeeding and formula feeding.

Study findings are consistent with others who have identified racial disparities in lactation and human milk feeding outcomes.^31–35,61^ A comparative, prospective observational study of 18,616 very low-birth-weight infants admitted to one of 134 NICUs in California revealed differences in feeding practices when outcomes were stratified by race and ethnicity.^32^ The authors attributed the differences to poorer quality of care provided to racial and ethnic minority populations.

In another study, family advocates and clinicians described patient neglect, stigmatization, and systemic barriers as factors that undermined quality, equitable NICU care.^63^ This study illustrated that interpersonal interactions with clinicians and families in the NICU, particularly around lactation support and human milk feeding, have a strong impact on infant feeding practices over the course of hospitalization and are associated with infant health outcomes both in the NICU and postdischarge.

Decisions about the use of PDHM in NICUs across the United States are commonly driven by physicians' care plans and institutional culture.^68^ At the study institution, as per the infant feeding protocol, all infants at <32 weeks adjusted gestational age in the NCCC are prioritized for an exclusive human milk diet, through a combination of MOM and PDHM, when it is available (S. Meier, NCCC, personal communication).

Feeding protocols such as this one may buffer against individual provider biases that hinder equitable access to PDHM. For example, they may minimize the influence of health care providers' assumptions about PDHM acceptability among culturally and racially diverse populations. They may also mitigate the negative consequences of stigma and discrimination, which inhibit effective communication for shared clinical decision-making.

However, starting at 32 weeks adjusted gestational age, the site protocol is to gradually decrease supplementation with PDHM and as MOM feeding increases. The care priority is to transition infants to exclusive breastfeeding or feeding with expressed MOM before discharge. If MOM is insufficient or not available during this inpatient care transition, formula rather than PDHM is often used.

A transition to exclusive MOM by the time of discharge for NICU-admitted infants involves a suite of coordinated care, including skilled lactation support, frequent skin-to-skin contact, milk expression when breastfeeding is hindered, and mental health and psychosocial support.^46,69^ It also is enhanced with culturally responsive education about the importance of exclusive human milk feeding in the first 6 months of an infant's life, information about the uses and safety of PDHM, risks of formula feeding, and respectful shared decision-making between the infant's caregivers and health care team.^39^

However, systemic racism and related structural barriers to quality perinatal care often constrain families' equitable participation in shared decision-making with members of the NICU care team.^15^ For example, employment obligations, lack of paid parental leave, and geographic distance are common barriers that may hinder parents' ability to be at their infant's bedside in the NICU, express milk and deliver it to their infant, or get access to early and continued lactation support during their infant's NICU stay.^33,39,70^

Patient and family-centered decision-making relies on effective communication, which may be hampered by language, educational attainment, and cultural differences, as well as stigma and discrimination associated with racism.^15^ In our study, after adjustment for insurance coverage, birth weight, gestational age at birth, mode of delivery, and language preference, racial–ethnic disparities persisted, suggesting that racial discrimination within postpartum care is a significant barrier to equitable lactation and infant feeding outcomes, particularly among non-Hispanic Black and Hispanic women.

Policies and protocols to expand PDHM supplementation to infants in postnatal units may be similarly ineffective in closing gaps in its access and utilization due to structural racism, institutional racism, and individual health care provider racial biases.^35,50,61,62^ Although well infants at NCWH may receive supplementation with PDHM when it is available, its use may depend on prenatal communication of this health care service, whether a health care provider offers it, or if a parent requests PDHM.

Implicit biases may be more pronounced in clinical scenarios such as these, when health care staff are responsible for initiating discussions about lactation and PDHM, providing patient education, and supporting shared decision-making for infant feeding.^54,71,72^ Other studies have shown that maternal preferences for breastfeeding and the use of PDHM are influenced by a number of complex factors, including religious, sociocultural, and historical influences, as well as perceived trustworthiness of health care providers, health care systems, and the safety of PDHM.^13,28,55–57,72,73^

Yet, even in situations where there are strong initial sociocultural barriers circumscribing acceptance of PDHM, research has found that patient-centered counseling and education about the importance of EHM feeding and PDHM positively influence maternal lactation outcomes and PDHM uptake, even among women who are reportedly less likely to find breastfeeding and PDHM acceptable.^47,69,74,75^

Our study contributes to the public health and medical literature at the intersection of birth equity and reproductive justice^6^; disparities and inequities in lactation cannot be understood separately from the context of pregnancy and birth.^76^ Dána-Ain Davis describes the confluence of medical racism and obstetric violence as “obstetric racism.”^9,17^ Widespread reports of racial and ethnic disparities in lactation support and use of PDHM across the literature demonstrate the effects of obstetric racism on lactation.

Further research is warranted to understand the knowledge, attitudes, and acceptability of lactation support and PDHM among culturally diverse populations in the United States.^57^ There is a need for community-engaged research that further examines the racialization of human milk, milk banking, and use of PDHM within and beyond the NICU setting.

Studies that document PDHM use by patient race/ethnicity, race/ethnicity of the health care team, geographic and place-based factors, the role of Baby-Friendly hospital practices, and other social determinants of health as part of continuous quality improvement efforts may help to identify institutional-specific barriers and facilitators of implementing recommended best practices in hospitals. There is also a need for clinical research that evaluates infant outcomes following supplementation with PDHM among term infants, which may improve the evidence base for expanding access to PDHM beyond the NICU.

Going forward, it will also be important to assess the impact of the COVID-19 pandemic, including the recent critical shortages of infant formula,^77^ on PDHM utilization by patient race and ethnicity during postpartum hospitalization. This pandemic has significantly impacted inpatient policies and practices and exacerbated perinatal health disparities and inequities.^78–80^

Additional research on the COVID-19 pandemic's impact on PDHM use will be critical to ensure equitable lactation support and resources are provided to mothers and families already marginalized and at risk for poor outcomes. As detailed by Elizabeth Howell et al, equity in care and outcomes can only be accomplished if it is a desired end in and of itself that is directly measured, monitored, and held accountable.^81^

### Limitations

The use of EMR data has limitations. We are limited in our analysis by the information that is recorded in the EMR. Although the EMR includes patient race, ethnicity, language, and insurance status, it does not permit us to assess patients' or health care providers' knowledge and attitudes about PDHM acceptability. Our analysis is also constrained by its retrospective nature and uneven distribution of patient records across relevant analytic categories.

For example, we are limited in our ability to interpret results for patients whose race–ethnicity is categorized as Other. This group may have also included infants in all of the other racial/ethnic groups, which could confound the interpretation of findings. The Asian and Pacific Islander group was also very small once we stratified by all modes of infant feeding, which limits the precision of our interpretation for this group.

Similarly, it is difficult to parse the importance of language in observed differences in PDHM use due to very small absolute numbers for each of the other languages documented within the Other category, precluding analysis.

The analyses would have been strengthened with quantification of the timing of introducing formula; relative proportion of MOM, PDHM, and formula; and duration of supplementation. Additionally, birth weight and gestational age may not sufficiently account for the length of stay and severity of illness, and these two variables are likely correlated with infant feeding.

In this article, we employ the terms “mother” and “maternal” to describe patients who delivered an infant at our institution. We also use “mother's own milk” and “breastfeeding” to describe modes of feeding. This language is consistent with information that was recorded in the EMRs and extracted to the Carolina Warehouse database.

We, however, acknowledge the limits of standardized heteronormative and binary sex, gender, and kinship descriptors in the EMR, and more broadly in sexual and reproductive health research,^82–85^ which obfuscate how the diversity of sex, gender, and family structure may affect reproductive health outcomes^86–88^ and perpetuate the continued erasure of gender-diverse childbearing populations in the United States.^89,90^

## Conclusions

This retrospective analysis of EMRs identified racial and ethnic disparities in the use of PDHM among non-Hispanic Black and Hispanic infants. The EMR is a powerful tool that can be used to identify patterns of inequitable health care that are shaped by structural, institutional, and interpersonal racism. Advancing patient-centered perinatal care includes strengthening readiness, recognition, response, reporting, and respect for antiracist and culturally responsive quality improvement.^91,92^

Policy changes, improving the racial and cultural diversity of teams who provide perinatal care and lactation support during hospitalization,^65,69,75,93^ may contribute to more equitable lactation outcomes and access to PDHM.

## Abbreviations Used

AAP: American Academy of Pediatrics
ABM: Academy of Breastfeeding Medicine
AWHONN: Association of Women's Health, Obstetric, and Neonatal Nurses
CI: confidence interval
EBF: exclusively breastfed
EMRs: electronic medical records
GEE: generalized estimating equations
HRSA: Health Resources and Services Administration
MOM: mother's own milk
NCCC: Neonatal Critical Care Center
NCWH: North Carolina Women's Hospital
NICUs: neonatal intensive care units
OR: odds ratio
PDHM: pasteurized donor human milk
WHO: World Health Organization
